# Synergy in the adulticidal efficacy of essential oils for the improvement of permethrin toxicity against *Aedes aegypti* L. (Diptera: Culicidae)

**DOI:** 10.1186/s13071-018-3001-7

**Published:** 2018-07-13

**Authors:** Arpaporn Chansang, Danita Champakaew, Anuluck Junkum, Atchariya Jitpakdi, Doungporn Amornlerdpison, Arunee Kongdee Aldred, Doungrat Riyong, Anchalee Wannasan, Jitrawadee Intirach, Roongtawan Muangmoon, Benjawan Pitasawat

**Affiliations:** 1 Center of Insect Vector Study, Department of Parasitology, Faculty of Medicine, Chiang Mai University, Chiang Mai, 50200 Thailand; 2 Graduate PhD’s Degree Program in Parasitology, Faculty of Medicine, Chiang Mai University, Chiang Mai, 50200 Thailand; 3 Faculty of Fisheries Technology and Aquatic Resources, Maejo University, Chiang Mai, 50290 Thailand; 4 Program in Chemistry, Faculty of Science, Maejo University, Chiang Mai, 50290 Thailand

**Keywords:** *Aedes aegypti*, *Cyperus rotundus*, *Alpinia galanga*, *Cinnamomum verum*, Synergy, Adulticidal, Essential oils, Permethrin

## Abstract

**Background:**

In a previous screening program for mosquitocides from local edible plants in Thailand, essential oils (EOs) of *Cyperus rotundus*, *Alpinia galanga* and *Cinnamomum verum*, were found to possess promising adulticidal activity against *Aedes aegypti*. With the aim of reducing usage of conventional insecticides and improving the management of resistant mosquito populations, this study was designed to determine the potential synergism in the adulticidal efficacy of EOs on permethrin toxicity against *Ae. aegypti*, both pyrethroid-resistant and -susceptible strains*.*

**Methods:**

EOs extracted from rhizomes of *C. rotundus* and *A. galanga* as well as *C. verum* barks were evaluated for chemical compositions and adulticidal activity against Muang Chiang Mai-susceptible (MCM-S) and Pang Mai Dang-resistant (PMD-R) strains of *Ae. aegypti*. Adulticidal bioassays of EO-permethrin mixtures for synergistic activity were also performed on these *Ae. aegypti* strains.

**Results:**

Chemical characterization by the GC-MS analytical technique demonstrated that 48 compounds were identified from the EOs of *C. rotundus*, *A. galanga* and *C. verum*, representing 80.22%, 86.75% and 97.24%, respectively, of all compositions. Cyperene (14.04%), *β*-bisabolene (18.27%) and cinnamaldehyde (64.66%) were the main constituents of *C. rotundus*, *A. galanga* and *C. verum* oils, respectively. In adulticidal bioassays, EOs of *C. rotundus*, *A. galanga* and *C. verum* were effective in killing *Ae. aegypti*, both MCM-S and PMD-R strains, with LD_50_ values of 10.05 and 9.57 μg/mg female, 7.97 and 7.94 μg/mg female, and 3.30 and 3.22 μg/mg female, respectively. The adulticidal efficacy against MCM-S and PMD-R *Ae. aegypti* of these EOs was close to that of piperonyl butoxide (PBO, LD_50_ values = 6.30 and 4.79 μg/mg female, respectively) but less pronounced than that of permethrin (LD_50_ values = 0.44 and 3.70 ng/mg female, respectively). Nevertheless, combination-based bioassays discovered the accomplished synergism of EOs together with permethrin. Significant synergistic effects with permethrin against both the strains of *Ae. aegypti* were recorded in the EOs of *C. rotundus* and *A. galanga*. Addition of *C. rotundus* and *A. galanga* oils decreased the LD_50_ values of permethrin against MCM-S dramatically from 0.44 to 0.07 and 0.11 ng/mg female, respectively, with synergism ratio (SR) values of 6.28 and 4.00, respectively. Furthermore, EOs of *C. rotundus* and *A. galanga* also reduced the LD_50_ values of permethrin against PMD-R drastically from 3.70 to 0.42 and 0.003 ng/mg female, respectively, with SR values of 8.81 and 1233.33, respectively.

**Conclusions:**

The synergy of enhanced adulticidal toxicity recorded from EO-permethrin combinations against both strains of *Ae. aegypti* presents a promising role of EOs as a synergist for improving mosquitocidal efficacy, particularly in situations where conventional compounds are ineffective or inappropriate.

## Background

The mosquito, *Aedes aegypti* L. (Diptera: Culicidae), is a dominant vector capable of transmitting dengue fever and other infectious viral diseases such as yellow fever, chikungunya and Zika, which pose a great and continued threat to mankind [1, 2]. Dengue virus is the most serious pathogenic hemorrhagic fever infecting humans, with an estimated 50–100 million cases each year and over 2.5 billion people at risk worldwide [3]. The outbreaks of this communicable disease inflict a huge burden on populations, health systems and economies in most tropical countries [1]. According to Thailand’s Ministry of Public Health, 142,925 dengue cases and 141 deaths were reported throughout the country in 2015, which is more than triple the number of cases and deaths in 2014 [4]. Although there is documented history of dengue fever being eliminated or significantly reduced through *Ae. aegypti* control [5], the infection rates have in fact risen drastically, with an explosive growth of illness worldwide, partly due to decades of global warming. The elimination and control of *Ae. aegypti* is relatively difficult because it is a domestic mosquito vector that mates, feeds, rests and lays eggs during daylight in and around human habitations. Furthermore, this mosquito has the ability to adapt to environmental changes or disturbances resulting from either natural phenomena, such as droughts, or human control measures, and it can bounce back to its initial population size [6, 7]. Since dengue vaccine has only been registered recently and no specific dengue therapeutics are available, preventing and reducing the risk of dengue transmission depends entirely on controlling mosquito vectors and interrupting human-vector contact.

Mosquito control by chemical utilization specifically, currently plays a role in public health as an essential part of comprehensive and integrated vector management. The most popular chemical methods involve application of insecticides with low toxicity that target mosquito larvae (larvicide) and adults (adulticide). Larval control by source reduction and routine application of chemical larvicides, such as organophosphate compounds and insect growth regulators, is considered as fundamental intervention. However, the adverse environmental impact associated with synthetic pesticides as well as their labor intensiveness and difficulty to sustain remain a major challenge [8, 9]. Conventional, aggressive vector management such as adulticide application remains the most efficient means of control during a virus outbreak, due to prompt and massive elimination of infective mosquito vectors as well as a reduction of the lifespan and total reproduction capacity of local vector populations [3, 10]. Four classes of chemical insecticides, namely organochlorines (exclusively DDT), organophosphates, carbamates and pyrethroids, are the mainstay of vector control programs, with pyrethroids considered as the most successful class, providing high potency against a wide variety of arthropods and low mammalian toxicity. Synthetic pyrethroids now constitute the majority of commercial insecticides and account for approximately 25% of the global insecticide market [11, 12]. Permethrin and deltamethrin are broad-spectrum pyrethroid insecticides that have been used worldwide for decades, controlling a wide range of insect pests of agricultural and medical importance [13, 14]. During the 1950s, DDT was the chemical of choice for national public health mosquito control programs in Thailand. After applying DDT extensively in endemic areas of malaria, its use was phased out gradually in Thailand between 1995 and 2000, and replaced by two pyrethroids: permethrin and deltamethrin [15, 16]. These pyrethroid insecticides were introduced in the early 1990s for controlling malaria and dengue, mainly by treating bednets as well as using thermal fogging and ULV sprays [14, 17]. However, they lost their effectiveness due to significant mosquito resistance and non-compliance from the community because of concern about public health and the environmental impacts of synthetic chemicals. This caused major problems that threatened the success of vector control programs [14, 18, 19]. Prompt and appropriate counter measures were required to improve the effectiveness of a strategy. Recommended management procedures included substitution by natural substances, rotation of different classes of chemicals, addition of a synergist, and mixing of chemicals or concurrent mosaic application of different classes of chemicals [14, 20, 21]. Therefore, the search for and development of an environmental and user friendly alternative and synergist with high efficacy are urgently needed, and this study aimed to satisfy this demand.

Insecticides of natural origin, especially those based on botanical components, have shown potential in assessing current and future mosquito control alternatives [22–24]. Several studies have demonstrated the possibility of using plant-based products, particularly essential oils (EOs) as adulticides for controlling important mosquito vectors. Adulticidal properties against certain important mosquito species have been identified in many plant oils such as *Apium graveolens*, *Carum carvi*, *Curcuma zedoaria*, *Illicium verum*, *Piper longum*, *Thymus vulgaris*, *Schinus terebinthifolia*, *Cymbopogon citratus*, *Cymbopogon schoenanthus*, *Cymbopogon giganteus*, *Chenopodium ambrosioides*, *Cochlospermum planchonii*, *Eucalyptus tereticornis*, *Eucalyptus citriodora*, *Cananga odorata* and *Petroselinum crispum* [25–30]. EOs are currently not only applied individually but also used in mixtures with extracted botanicals or existing synthetic insecticides, generating varied degrees of toxicity. A combination of conventional insecticides in the class of organophosphates, carbamates and pyrethroids with EOs/plant extracts acts synergistically or antagonistically in toxic efficacy and has been documented as effective against disease vectors and pests [31–35]. However, most studies on the synergistically toxic effects of combinations involving phytochemicals, with and without synthetic chemicals, have been conducted in agricultural insect vectors and pests rather than mosquitoes of medical importance. Furthermore, much work on the synergistic activity of botanical-synthetic insecticide combinations against mosquito vectors has focused on larvicidal action.

In a previous study by the authors, as part of an ongoing research project for screening adulticides from local edible plants in Thailand, EOs of *Cyperus rotundus*, *Alpinia galanga* and *Cinnamomum verum* were found to possess potential adulticidal activity against *Ae. aegypti* [36]. This study was, therefore, designed to evaluate the adulticidal efficacy of EOs isolated from these medicinal plants against *Ae. aegypti*, both pyrethroid resistant and susceptible strains. The synergistic effect of binary mixtures of EOs and synthetic pyrethroids, with promising adulticidal relevance, was also assayed with the aim of reducing the use of conventional insecticides and improving the management of resistant mosquito vectors, specifically *Ae. aegypti*. In this paper, the chemical profiles of effective EOs and their potential for enhancing the toxicity of synthetic permethrin were reported against *Ae. aegypti* in both pyrethroid susceptible (MCM-S) and resistant (PMD-R) strains.

## Methods

### Plant materials

**Fig. 1:**
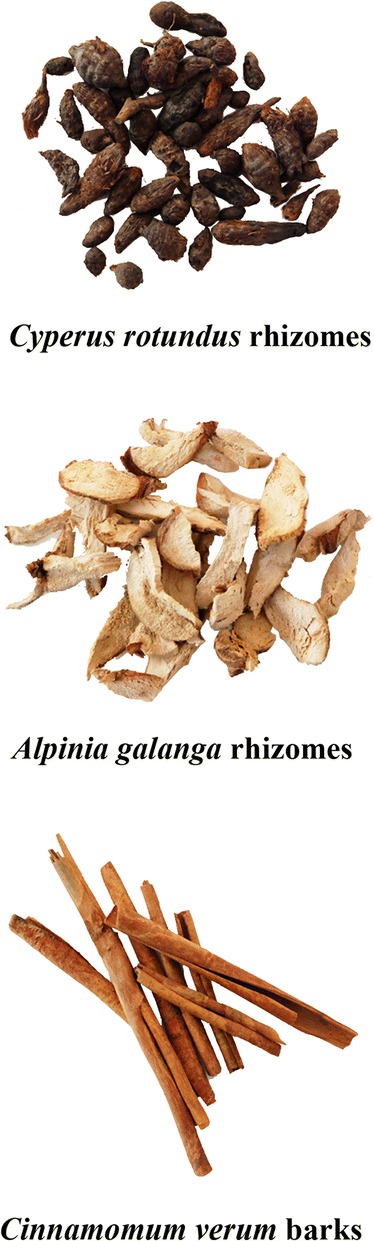
Dried plant materials of *Cyperus rotundus*, *Alpinia galanga* and *Cinnamomum verum*

Rhizomes of *C. rotundus* and *A. galanga* as well as *C. verum* barks (Fig. 1) used for extraction of essential oils were obtained commercially from herb suppliers in Chiang Mai Province, Thailand. Scientific identification of these plants was accomplished by consulting Mr James Franklin Maxwell, a botanist at the Chiang Mai University (CMU) Herbarium, Department of Biology, Faculty of Science, CMU, Chiang Mai Province, Thailand; and Miss Wannaree Charoensup, a scientist at the Department of Pharmaceutical Science, Faculty of Pharmacy, CMU. A voucher specimen of each plant was deposited for future reference at the Department of Parasitology, Faculty of Medicine, CMU.

### Extraction of essential oils

The plant specimens were shade-dried separately for 3–5 days in an open area, with active ventilation and ambient temperature of about 30 ± 5 °C, in order to remove the moisture content prior to extracting the natural essential oils (EOs). A total of 250 g of each dried plant material was pulverized mechanically to a coarse powder and used to isolate essential oil (EO) by steam distillation. The distillation apparatus consisted of an electric heating mantle, a 3000 ml round-bottomed flask, an extraction column, a condenser and a cool ace (Eyela Cool Ace CA-1112 CE, Tokyo Rikakikai Co. Ltd., Tokyo, Japan). The flask was filled with 1600 ml of distilled water and 10–15 glass beads, which was then heated by an electric heating mantle to about 100 °C for at least 3 h until completion of distillation, after which no more EO could be obtained. The EO layer was separated from the water phase using a separatory funnel, dried over anhydrous sodium sulfate (Na_2_SO_4_) and preserved in an airtight brown bottle at 4 °C until required for investigating the chemical composition and adulticidal activity.

### Gas chromatography-mass spectrometry (GC-MS) for EO analysis

The chemical constituents of EOs were analyzed in parallel with the adulticidal bioassays. Qualitative analyses were performed by using the GC-MS system comprising a Hewlett-Packard (Wilmington, CA, USA) 7890A gas chromatographer, equipped with a single quadrupole mass selective detector (Agilent Technologies, Wilmington, CA, USA) and an MSD 5975C (EI) (Agilent Technologies).

The column was a DB-5MS (30 m × 0.25 mm ID × 0.25 μm film thickness). The total GC-MS running time was 20 min. The analytical conditions were injector and transfer line temperatures of 250 and 280 °C, respectively; the oven temperature was programmed to increase from 50 to 250 °C at 10 °C/min; the carrier gas was helium at 1.0 ml/min; the injection was of 0.2 μl (1/10% v/v, in CH_2_Cl_2_); and the split ratio was at 100:1. An electron ionization system with an ionization energy of 70 eV was used for the GC-MS detection. The data were acquired over a range of 50–550 atomic mass units (amu) with a scan rate of 2.91 scans/s. The relative percentages of the constituents were expressed as percentage by peak area normalization. Identification of the components in the EOs was based on their retention indices (RI). The RI was calculated by the equation of Van den Dool & Kratz [37] in relation to a homologous series of *n*-alkanes (C_8_-C_40_) and compared with the retention indices of the literature [38] and with the library’s databases (NIST 2008 and Wiley 8NO8). The identity of the indicated compounds such as structures and the molecular formulae were confirmed by comparison with available authentic samples.

### Chemicals

The analytical standard of synthetic permethrin and piperonyl butoxide (PBO, a positive control in the synergist study) was purchased from Sigma-Aldrich (St. Louis, MO, USA). World Health Organization (WHO) adult test kits and the diagnostic dose of permethrin-impregnated papers (0.75%) were obtained commercially from the WHO Vector Control Unit, Penang, Malaysia. All other chemicals and reagents used were of analytical grade and were procured from local agencies in Chiang Mai province, Thailand.

### Test mosquitoes and maintenance

The mosquitoes used as the test organism in the adulticidal bioassays were free-mating laboratory *Ae. aegypti*, including Muang Chiang Mai-susceptible (MCM-S) and Pang Mai Dang-resistant (PMD-R) strains. The MCM-S strain was initiated from local specimens collected at Muang Chiang Mai District, Chiang Mai Province, Thailand, and had been maintained consecutively since 1995 in the insectary of the Department of Parasitology, Faculty of Medicine, CMU [39]. The PMD-R strain, which proved to be resistant to permethrin, was established from the field mosquitoes collected originally at Ban Pang Mai Dang, Mae Tang District, Chiang Mai Province, Thailand, and had been maintained in the same institute since 1997 [40]. The PMD-R strain was reared under selection pressure for maintaining the resistance level by regular exposure to 0.75% permethrin, using WHO test kits with some modifications [41]. Each strain of *Ae. aegypti* was colonized separately in a laboratory free of exposure to pathogens at 25 ± 2 °C and 80 ± 10% RH under a 14:10 h light/dark photoperiod cycle. Approximately 200 larvae were reared in plastic trays (33 cm long × 28 cm wide × 9 cm high) containing tap water at densities in the range of 150–200 larvae/tray, and fed on sterilized ground dog-biscuits twice daily. Adults were reared in humidified cages and given continuous access to 10% aqueous sucrose and 10% multivitamin syrup solution. Female mosquitoes were blood-fed periodically for egg production. Two- to five-day-old non-blood-fed females were available continuously for experimental adulticidal bioassays.

### Adulticidal bioassays of EOs

The dose-mortality response bioassays of EOs were carried out against adult female *Ae. aegypti*, MCM-S and PMD-R strains, by using the topical application method modified from the standard protocol of WHO susceptibility test [42]. The EO of each plant was diluted serially in a suitable solvent such as ethanol or acetone in order to prepare a graded series of 4–6 concentrations. The mosquitoes were weighed individually after being anaesthetized with carbon dioxide (CO_2_). The anaesthetized mosquitoes were subsequently kept immobile over dry filter paper on a custom cold plate standing under a stereomicroscope for preventing reanimation during manipulations. For each treatment, a 0.1 μl volume of EO solution was applied to the upper part of the female’s pronotum by using a handheld Hamilton microdispenser (700 series Microliter™, Hamilton Company, Reno, NE, USA). A group of 25 individual females was treated at each concentration, with at least 4 different concentrations covering the range of mortality from 10 to 95%. The solvent-treated mosquitoes served as the control group. In order to prevent contamination among the test samples, the filter paper was replaced with a new one for each EO tested. The dosages used in these bioassays were expressed in μg of EO per mg of body weight of a live female. The adulticidal activity of PBO, which was used in a synergism experiment as the positive control, was also evaluated in a manner similar to that for the EOs. The treated mosquitoes in all the groups were transferred into plastic cups and provided with 10% sucrose plus 10% multivitamin syrup. All bioassays were carried out at 25 ± 2 °C and 80 ± 10% RH, and replicated four times together with the control. Mortality at a 24 h holding period was checked and confirmed by the lack of mosquito response to mechanical stimuli, and then reported from the average of four replicates. Four experimental treatments were repeated for each test sample by using different batches of mosquitoes. The results obtained were pooled for calculating the mortality percentages used in the determination of 24 h lethal dosages by the Probit analysis.

### Adulticidal bioassays of EO-permethrin mixtures

Evaluation of EOs for their synergistically adulticidal effects with permethrin was performed by using the topical toxicity assay procedure [42], as described earlier. Acetone or ethanol was used as the solvent for preparing the required concentrations of permethrin as well as binary mixtures of EO and permethrin (EO-permethrin: permethrin mixed with an LD_25_ concentration of EO). Groups of the test substances, permethrin and EO-permethrin, were evaluated against both MCM-S and PMD-R strains of *Ae. aegypti*. Four doses of permethrin were applied to each of 25 female mosquitoes for testing their adulticidal efficacy, and each treatment was replicated four times. Four to six doses of EO-permethrin were applied to each of the 25 female mosquitoes for identifying synergist EO candidates, and each application was replicated four times. The PBO-permethrin treatment (permethrin mixed with an LD_25_ concentration of PBO) also was included as the positive control. The dosages used in these bioassays were expressed in ng of the test sample per mg of body weight of a live female. Four experimental assessments against each mosquito strain were accomplished from separately reared batches, and the mortality data were pooled and analyzed by Probit to determine 24 h lethal dosages.

### Statistical analysis

The percentage mortality was corrected by Abbott’s formula [43]. The corrected data were analyzed by Probit regression analysis using the computerized statistical program SPSS (Version 19.0). The lethal values of 25%, 50%, 90%, 95% and 99% (LD_25_, LD_50_, LD_90_, LD_95_ and LD_99_, respectively) were calculated with corresponding 95% confidence intervals (95% CI). The Chi-square test or the Mann-Whitney U-test was used in each bioassay for the assessment of significance and the measurement of difference between the test samples. Statistically significant results were considered at *P* < 0.05. The resistance ratio (RR) was estimated at the LD_50_ level by using the following formula [12]:

$$ \mathrm{RR}={\mathrm{LD}}_{50}\ \mathrm{of}\ \mathrm{resistant}\ \mathrm{strain}/{\mathrm{LD}}_{50}\ \mathrm{of}\ \mathrm{susceptible}\ \mathrm{strain} $$

A value of RR > 1 indicates resistance and RR ≤ 1 indicates susceptible. The synergism ratio (SR) values of each candidate synergist were calculated as follows [34, 35, 44]:

$$ \mathrm{SR}={\mathrm{LD}}_{50}\ \mathrm{of}\ \mathrm{permethrin}\ \mathrm{with}\mathrm{out}\ \mathrm{synergist}/{\mathrm{LD}}_{50}\ \mathrm{of}\ \mathrm{permethrin}\ \mathrm{with}\ \mathrm{synergist} $$

This factor differentiates and classifies the results into three categories: an SR value of 1 ± 0.05 is considered as no obvious effect, an SR value > 1.05 is considered as synergistic, and an SR value < 0.95 is considered as antagonism.

## Results

### Chemical compositions of EOs

**Fig. 2:**
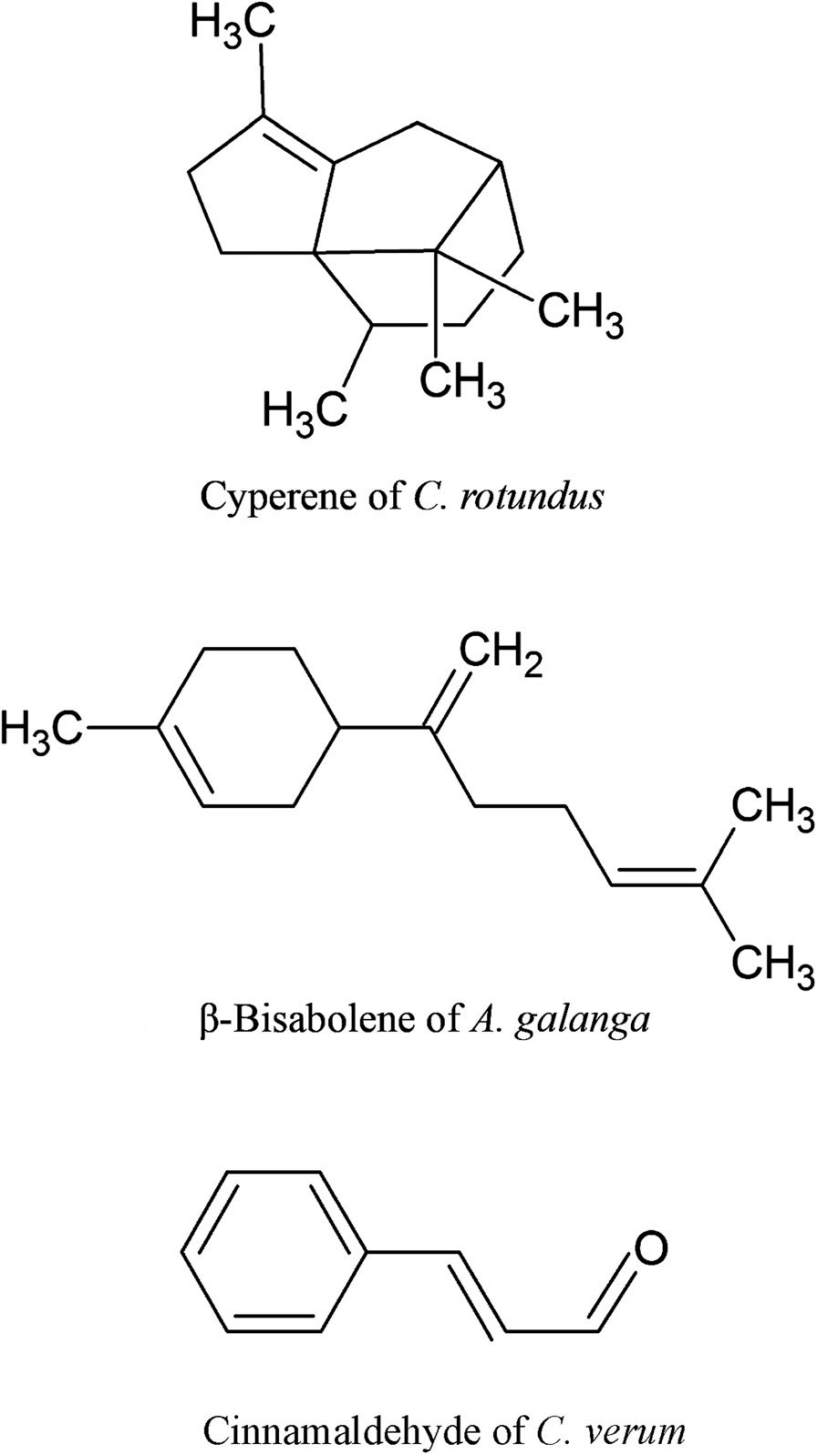
Chemical structures of the main constituents in the EOs of *C. rotundus*, *A. galanga* and *C. verum*

The steam distillations of *C. rotundus* and *A. galanga* rhizomes as well as *C. verum* barks provided pale yellow liquid oils, with yields of 0.15, 0.27 and 0.54% (v/w) on dry weight basis, respectively (Table 1). GC-MS studies on the chemical constituents of *C. rotundus*, *A. galanga* and *C. verum* oils revealed the presence of 19, 17 and 21 compounds, constituting 80.22, 86.75 and 97.24% of all the compositions, respectively (Table 2). Compounds in the rhizome oil of *C. rotundus* comprised mostly cyperene (14.04%), followed by calamenene (9.57%), α-copaene (7.97%) and α-cubebene (7.53%). The chief chemical constituent of *A. galanga* rhizome oil was *β*-bisabolene (18.27%), followed by α-bergamotene (16.28%), 1,8-cineole (10.17%) and chavicol (10.09%). While cinnamaldehyde (64.66%) was identified as the major component of *C. verum* bark oil, cinnamyl acetate (6.61%), α-copaene (5.83%) and 3-phenylpropanal (4.09%) were seen as the minor constituents. Chemical structures of cyperene, *β*-bisabolene and cinnamaldehyde, which were the main compounds of *C. rotundus*, *A. galanga* and *C. verum*, respectively, are displayed in Fig. 2.

**Table 1:** Physical characteristics and percentage yield of essential oils extracted from three plant species

Family/species	Common name	Voucher specimen	Part used	Physical characteristics		Yield (%)
				Color	Density (g/ml)	
Cyperaceae						
*Cyperus rotundus*	Nut grass	PARA-CY-001-Rh/3	Rhizome	Pale yellow	0.96	0.15
Zingiberaceae						
*Alpinia galanga* var. galanga	Galanga	PARA-AL-001-Rh/3	Rhizome	Pale yellow	0.89	0.27
Lauraceae						
*Cinnamomum verum*	Ceylon cinnamon	PARA-CI-007-Ba/3	Bark	Pale yellow	1.02	0.54

**Table 2:** Chemical composition of plant essential oils

No.	Chemical constituent	RI_Calc_^a^	RI_Lit_^b^	Percentage composition (%)		
				*C. rotundus*	*A. galanga*	*C. verum*
1	*α*-Pinene	940	944		0.78	0.74
2	Camphene	957	961			0.47
3	Benzaldehyde	967	971			1.14
4	*β*-Pinene	983	985		1.40	0.34
5	Limonene	1033	1035		1.24	0.74
6	1,8-Cineole	1039	1040		10.17	1.36
7	Linalool	1100	1111			0.47
8	3-Phenylpropanal	1168	1170			4.09
9	Borneol	1180	1181			1.13
10	Terpinen-4-ol	1186	1188			1.18
11	*Α*-Terpineol	1202	1211			2.45
12	Myrtenol	1202	1212	2.36		
13	Cinnamaldehyde	1225	1305			64.66
14	Chavicol	1345	1346		10.09	
15	*α*-Cubebene	1353	1355	7.53		0.36
16	Geranyl acetate	1376	1377		1.84	
17	*α*-Copaene	1385	1386	7.97		5.83
18	*β*-Elemene	1395	1395		2.76	
19	*α*-Guaiene	1395	1396	2.73		
20	Methyl eugenol	1399	1407		2.17	
21	Cyperene	1418	1419	14.04		
22	*α*-Santalene	1425	1426		1.13	
23	*β*-Caryophyllene	1431	1432	1.18		1.63
24	Cinnamic acid	1436	1438			0.46
25	α-Bergamotene	1441	1442		16.28	
26	Cinnamyl acetate	1450	1452			6.61
27	*β*-Farnesene	1453	1454		4.55	
28	*cis*-9-Dodecenyl acetate	1457	1459	3.34		
29	*γ*-Muurolene	1483	1483	3.16		
30	*α*-Curcumene	1485	1485		1.14	
31	Pentadecane	1503	1503		7.50	
32	*α*-Muurolene	1506	1506	1.93		
33	*δ*-Guaiene	1511	1511	2.22		
34	*β*-Bisabolene	1516	1517		18.27	
35	*δ*-Cadinene	1524	1525	2.37		1.15
36	L-Calamenene	1530	1531			0.66
37	*β*-Sesquiphellandrene	1532	1533		5.37	
38	Calamenene	1533	1534	9.57		
39	4b,5,6,7,8,8a,9,10-Octahydro-1-methylphenanthrene	1543	1544	2.55		
40	4,7-Dimethyl-1-tetralone	1558	1559	0.95		
41	*β*-Caryophyllene epoxide	1596	1597	4.90		1.39
42	Cadina-1,4-diene	1637	1638			0.38
43	Ledene	1668	1669	3.39		
44	8-Heptadecene	1675	1676		0.91	
45	Cadalene	1683	1683	2.95		
46	Oxo-*α*-Ylangene	1687	1694	1.20		
47	Isopetasan	1714	1715	5.88		
48	Farnesyl acetate	1831	1831		1.15	
	Total identified			80.22	86.75	97.24
	No. of identified constituents			19	17	21

^a^Retention indices are determined using *n*-alkanes (C_8_-C_40_)

^b^Retention indices of reference compounds from literature

### Adulticidal efficacy of EOs on *Ae. aegypti*

The results of three EOs evaluated for adulticidal activity against *Ae. aegypti* are shown in Table 3. It was found that all of the EOs exhibited lethal effects, varying in type and dosage against MCM-S *Ae. aegypti*. The most effective EO was *C. verum*, followed by *A. galanga* and *C. rotundus*, with LD_50_ values of 3.30, 7.97 and 10.05 μg/mg MCM-S female, respectively, which were insignificantly higher than those of 3.22 (*U* = 1, *Z* = -0.775, *P =* 0.667), 7.94 (*U* = 2, *Z* = 0, *P* = 1) and 9.57 (*U* = 0, *Z* = -1.549, *P* = 0.333) μg/mg PMD-R female, respectively. This corresponded relatively to PBO, which offered slightly higher adulticidal effects on PMD-R rather than MCM-S strains, with LD_50_ values of 4.79 and 6.30 μg/mg female, respectively (*U* = 0, *Z* = -2.021, *P* = 0.057). It can be estimated that the LD_50_ values of *C. verum*, *A. galanga*, *C. rotundus* and PBO against PMD-R were approximately 0.98-, 0.99-, 0.95- and 0.76-fold lower than those against MCM-S, respectively. Therefore, this indicated the relative similarity in PBO and EO susceptibility between the two strains of *Ae. aegypti* although PMD-R was insignificantly more susceptible than MCM-S. In contrast, susceptibility to permethrin was vastly different between these two strains of *Ae. aegypti* (Table 4). PMD-R displayed significant resistance to permethrin as expected when compared to MCM-S (LD_50_ value = 0.44 ng/mg female), with a higher LD_50_ value of 3.70 ng/mg female (*U* = 0, *Z* = -2.309, *P* = 0.029). Although PMD-R was far less susceptible to permethrin than MCM-S, its susceptibility to PBO as well as *C. verum*, *A. galanga* and *C. rotundus* oils was slightly higher than that in MCM-S.

**Table 3:** Adulticidal activity of EOs and PBO against pyrethroid susceptible (MCM-S) and resistant (PMD-R) strains of *Ae. aegypti*

Essential oil dosage (μg/mg female)	%mortality (mean ± SE)	Adulticidal activity (95% CI, μg/mg female)			*χ* ^*2*^	*df*	SE	Regression coefficient
		LD_25_	LD_50_	LD_90_				
*Cyperus rotundus*								
MCM-S		6.12	10.05	17.52	0.34	3	0.16	6.22
4.80	17.00 ± 1.58	(4.95–6.99)	(9.37–10.78)	(16.00–19.76)				
6.72	29.00 ± 1.66							
9.60	48.50 ± 2.16							
11.52	60.50 ± 1.55							
14.40	76.00 ± 1.41							
PMD-R		5.82	9.57	16.70	1.02	3	0.27	6.48
4.80	18.00 ± 0.76	(4.66–6.68)	(8.91–10.25)	(15.34–18.68)				
6.72	34.00 ± 1.07							
9.60	48.00 ± 1.51							
11.52	63.00 ± 1.16							
14.40	81.50 ± 2.07							
*Alpinia galanga*								
MCM-S		6.49	7.97	10.77	0.12	2	0.66	16.85
7.12	34.50 ± 1.06	(5.64–6.98)	(7.61–8.25)	(10.18–11.81)				
8.01	52.00 ± 1.06							
8.90	65.50 ± 1.18							
9.79	80.00 ± 1.41							
PMD-R		6.80	7.94	10.12	0.84	2	1.76	20.62
7.12	30.00 ± 1.41	(6.25–7.16)	(7.67–8.17)	(9.72–10.74)				
8.01	55.00 ± 0.71							
8.90	69.00 ± 0.71							
9.79	86.50 ± 1.85							
*Cinnamomum verum*								
MCM-S		2.58	3.30	4.66	5.02	3	2.51	32.16
2.55	23.00 ± 1.58	(2.33–2.77)	(3.16–3.43)	(4.44–4.98)				
3.06	44.50 ± 2.29							
3.57	61.00 ± 2.92							
4.08	69.50 ± 1.59							
4.59	92.50 ± 2.23							
PMD-R		2.50	3.22	4.59	1.96	3	0.45	31.67
2.55	28.00 ± 1.31	(2.24–2.70)	(3.08–3.35	(4.37–4.89)				
3.06	44.00 ± 0.93							
3.57	61.00 ± 1.16							
4.08	75.50 ± 0.99							
4.59	93.00 ± 2.25							
PBO								
MCM-S		1.63	6.30	15.18	1.67	3	0.66	5.2
4.24	34.50 ± 1.63	(-1.02–3.21)	(5.14–7.15)	(13.52–17.97)				
6.36	53.50 ± 1.84							
8.48	65.50 ± 2.33							
10.60	72.75 ± 2.04							
12.72	80.50 ± 1.20							
PMD-R		0.70	4.79	12.55	0.69	2	0.80	5.81
4.24	44.25 ± 2.70	(-2.76–2.48)	(3.24–5.72)	(11.01–15.50)				
6.36	63.50 ± 1.75							
8.48	72.75 ± 2.90							
10.60	82.25 ± 1.96

**Table 4:** Adulticidal activity of permethrin and its combinations with EO or PBO against pyrethroid susceptible (MCM-S) and resistant (PMD-R) strains of *Ae. aegypti*

Chemical/mosquito	%mortality	Adulticidal activity (95% CI, ng/mg female)			*χ2*	*df*	SE	RC	SR	Effect
(ng/mg female)	(mean ± SE)	LD_50_	LD_95_	LD_99_						
Permethrin (PE)										
MCM-S		0.44	0.76	0.89	0.52	2	0.62	5.17	ND	ND
0.3	22.25 ± 1.36	(0.41–0.46)	(0.69–0.86)	(0.80–1.03)						
0.4	44.75 ± 0.91									
0.5	60.75 ± 0.40									
0.6	79.75 ± 0.85									
PMD-R		3.7	10.41	13.19	0.76	2	0.03	0.24	ND	ND
2	31.75 ± 1.48	(3.01–4.25)	(9.18–12.40)	(11.43–16.08)						
4	56.50 ± 2.63									
6	70.75 ± 1.70									
8	84.75 ± 1.36									
PE+*C. rotundus* (LD_25_)										
MCM-S		0.07	0.28	0.36	4.36	3	0.94	7.99	6.28	Synergistic
0.0125	25.25 ± 1.89	(0.06–0.09)	(0.24–0.34)	(0.31–0.45)						
0.025	35.75 ± 1.95									
0.05	49.50 ± 2.47									
0.1	61.75 ± 2.03									
0.2	82.50 ± 1.01									
PMD-R		0.42	2.03	2.7	8.55	4	0.1	1.02	8.81	Synergistic
0.125	29.25 ± 0.60	(0.09–0.65)	(1.62–2.90)	(2.12–3.96)						
0.25	42.50 ± 0.81									
0.5	63.50 ± 1.45									
1	75.00 ± 1.25									
1.5	85.75 ± 1.41									
2	92.75 ± 2.23									
PE+*A. galanga* (LD_25_)										
MCM-S		0.11	0.44	0.57	2.15	2	0.6	5.09	4	Synergistic
0.0625	35.00 ± 1.25	(0.08–0.14)	(0.38–0.52)	(0.49–0.70)						
0.125	57.00 ± 1.44									
0.25	76.75 ± 0.83									
0.5	96.75 ± 5.40									
PMD-R		0.003	0.015	0.021	5.51	2	17.88	135.02	1,233.33	Synergistic
0.0015625	33.50 ± 1.20	(-0.031–0.008)	(0.010–0.197)	(0.013–0.290)						
0.003125	53.50 ± 1.15									
0.00625	71.75 ± 2.11									
0.0125	86.50 ± 1.09									
PE+*C. verum* (LD_25_)										
MCM-S		0.42	0.89	1.08	0.88	3	0.43	3.53	1.05	No effect
0.2	22.25 ± 1.78	(0.39–0.46)	(0.79–1.05)	(0.95–1.30)						
0.3	34.00 ± 2.39									
0.4	45.75 ± 2.34									
0.5	57.50 ± 1.63									
0.6	76.00 ± 1.83									
PMD-R		3.85	11.04	14.01	0.38	2	0.03	0.23	0.96	No effect
2	32.00 ± 2.42	(3.14–4.43)	(9.64–13.36)	(12.03–17.36)						
4	53.25 ± 2.82									
6	69.75 ± 3.29									
8	81.75 ± 3.42									
PE+PBO (LD_25_)										
MCM-S		0.26	0.68	0.86	0.65	2	0.61	3.86	1.69	Synergistic
0.2	41.00 ± 1.84	(0.21–0.29)	(0.60–0.84)	(0.74–1.10)						
0.3	55.00 ± 2.18									
0.4	73.75 ± 2.28									
0.5	81.00 ± 1.95									
PMD-R		0.65	1.41	1.72	1.76	2	0.3	2.17	5.69	Synergistic
0.4	26.25 ± 1.59	(0.58–0.71)	(1.24–1.68)	(1.50–2.11)						
0.6	51.00 ± 2.14									
0.8	63.25 ± 1.91									
1	76.00 ± 2.28									

*Abbreviations*: RC, regression coefficient, SR, synergistic ratio, ND, not determined

### Synergistic activity of EO-permethrin mixtures

**Fig. 3:**
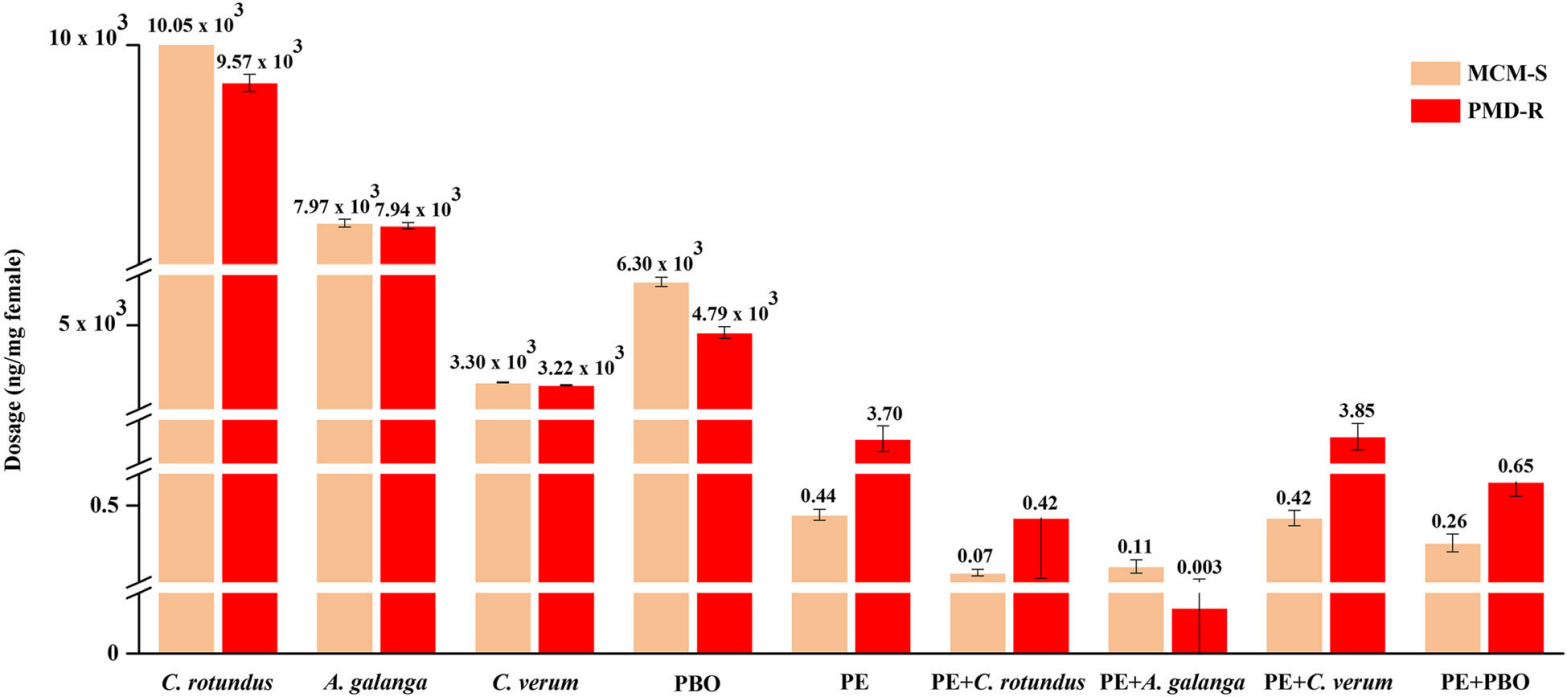
Adulticidal activity (LD_50_) of the EOs, PBO, permethrin (PE) and their combinations against the pyrethroid susceptible (MCM-S) and resistant (PMD-R) strains of *Ae. aegypti*

Binary mixtures of permethrin and EO (LD_25_) exhibited either synergism (SR value > 1.05) or no effect (SR value = 1 ± 0.05), as observed in adulticidal bioassays of EO-permethrin combinations. The combined adulticidal effects of EO-permethrin mixtures on the experimental *Ae. aegypti* of both MCM-S and PMD-R strains are shown in Table 4 and Fig. 3. The addition of *C. verum* oil was found to decrease the LD_50_ of permethrin slightly against MCM-S, and increase it slightly against PMD-R from 0.44 to 0.42 ng/mg female and 3.70 to 3.85 ng/mg female, respectively. By contrast, adding oils of *C. rotundus* and *A. galanga* vastly diminished the LD_50_ of permethrin against MCM-S from 0.44 to 0.07 (*U* = 0, *Z* = -2.309, *P* = 0.029) and to 0.11 (*U* = 0, *Z* = -2.309, *P* = 0.029) ng/mg female, respectively. The SR values of EO-permethrin mixtures, determined from the LD_50_ values against MCM-S were 6.28 and 4.00 with *C. rotundus* and *A. galanga* oils added, respectively. Correspondingly, the LD_50_ of permethrin declined greatly against PMD-R when *C. rotundus* and *A. galanga* oils were added, from 3.70 to 0.42 (*U* = 0, *Z* = -2.309, *P* = 0.029) and to 0.003 (*U* = 0, *Z* = -2.337, *P* = 0.029) ng/mg female, respectively. The SR value of permethrin coupled with *C. rotundus* against PMD-R was 8.81, whereas those of *A. galanga*-permethrin mixture was 1,233.33. Permethrin toxicity was also increased by the positive control, PBO, through decreasing of its LD_50_ values from 0.44 to 0.26 ng/mg female and 3.70 to 0.65 ng/mg female against MCM-S (*U* = 0, *Z* = -2.309, *P* = 0.029) and PMD-R (*U* = 0, *Z* = -2.309, *P* = 0.029), respectively. The SR values of PBO-permethrin mixtures were 1.69 and 5.69 against MCM-S and PMD-R strains, respectively. These findings suggest that the oils of *C. rotundus* and *A. galanga*, as well as PBO, synergized permethrin toxicity more than *C. verum* oil on both MCM-S and PMD-R strains.

## Discussion

Permethrin is an insecticide in broad-spectrum pyrethroid insecticides, which are a large class of structurally very diverse, synthetic analogues of natural pyrethrins discovered from flowers of pyrethrums (*Chrysanthemum* spp.) [45]. Synthetic pyrethroids are used worldwide to control virtually all arthropods of agricultural and medical importance. However, the need to reduce the use of conventional synthetics and develop alternatives is now urgent due to the deleterious effect of applying synthetic insecticides, particularly regarding developing and widespread mosquito resistance as well as the impact on long-term health and the environment [35, 46, 47]. In addition to protecting the environment and human health, the benefits of botanical insecticides are, for example, high selectivity, worldwide availability, and convenient production and application, which make them more attractive candidates for use in mosquito control management [32, 48, 49]. In this study, apart from GC-MS analysis for illustrating the chemical profiles of effective EOs, evaluation of the adulticidal activity of EOs and their potential for increasing the toxicity of synthetic permethrin were undertaken against *Ae. aegypti* in both pyrethroid susceptible (MCM-S) and resistant (PMD-R) strains.

GC-MS characterization demonstrated cyperene (14.04%), *β*-bisabolene (18.27%), and cinnamaldehyde (64.66%) as the principal constituents of *C. rotundus*, *A. galanga* and *C. verum* oils, respectively. These chemicals have demonstrated several biological activities. Ahn et al. [50] reported 6-acetoxy cyperene isolated from *C. rotundus* rhizomes as an anti-tumor compound that causes caspase-dependent apoptosis in ovarian cancer cells. *β*-bisabolene extracted from EO of *Commiphora guidottii* exhibited specific cytotoxicity to both human and murine mammary tumour cells *in vitro* and *in vivo* [51]. Cinnamaldehyde obtained from either natural extracts or synthesized in a laboratory has been reported to display insecticidal, antimicrobial, antifungal, anti-inflammatory, immunomodulatory, anticancer and anti-angiogenic activities [52].

The results obtained from the dose-dependent adulticidal bioassays demonstrated promising potential of the tested EOs and suggest similar susceptibility to EO and PBO in MCM-S and PMD-R strains of *Ae. aegypti*. The comparison of efficacy between EO and permethrin demonstrates that the latter had a much stronger adulticidal effect, with LD_50_ values of 0.44 and 3.70 ng/mg female against MCM-S and PMD-R strains, respectively. These findings corroborate with those of many studies in that insecticides of natural origin, particularly plant-derived products, are generally less effective than synthetic substances [31, 34, 35, 53, 54]. This is possibly due to the former being complex combinations of either active or inactive ingredients, whereas the latter is a purified single active compound. However, the variety and complexity of naturally active principles with different modes of action could be beneficial in either enhancing the bioactivity or impeding the evolution of resistance in vector populations [55–57]. The anti-mosquito potential of *C. verum*, *A. galanga* and *C. rotundus* as well as their constituents such as *β*-bisabolene, cinnamaldehyde and 1,8-cineole has been reported by many researchers [22, 36, 58–64]. However, a literature survey showed that there has been no previous report on their synergistic effects with permethrin or other synthetic insecticides against *Ae. aegypti.*

The susceptibilities to permethrin observed in this study were significantly different between the two strains of *Ae. aegypti*. While MCM-S was susceptible to permethrin, PMD-R showed far less susceptibility to it, with a resistance ratio of 8.41. The lower sensitivity to permethrin but higher sensitivity to EO of PMD-R, when compared to the sensitivity of MCM-S, opens the possibility for further investigation aimed at improving permethrin efficacy by combining it with EOs. Combination-based adulticidal bioassays for synergism found that binary mixtures of EO and permethrin produced either decreased or increased adult mortality in *Ae. aegypti*. Adding *C. verum* oil decreased the LD_50_ of permethrin slightly against MCM-S, but increased it slightly against PMD-R, with SR values of 1.05 and 0.96, respectively. This suggests that *C. verum* oil had no synergistic or antagonistic effect on permethrin when tested against both MCM-S and PMD-R. In contrast, the oils of *C. rotundus* and *A. galanga* showed significant synergistic effect by markedly minimizing the LD_50_ values of permethrin against either MCM-S or PMD-R. The SR values of EO-permethrin mixtures against MCM-S were 6.28 and 4.00 when permethrin combined with EOs of *C. rotundus* and *A. galanga*, respectively. Furthermore, a noticeable increment in the SR values was recorded when permethrin coupled with *C. rotundus* (SR = 8.81) or *A. galanga* (SR = 1233.33) was evaluated against PMD-R. It is worth noting that both *C. rotundus* and *A. galanga* potentiated the toxicity of permethrin against PMD-R *Ae. aegypti* significantly. Similarly, PBO was found to increase the permethrin toxicity, with SR values of 1.69 and 5.69 against MCM-S and PMD-R strains, respectively. As *C. rotundus* and *A. galanga* had the highest SR values, they were considered the best synergists in potentiating permethrin toxicity against MCM-S and PMD-R, respectively.

The synergistic effects of the combinations between synthetic insecticides and botanical extracts against various species of mosquitoes have been reported in several previous studies. Larvicidal bioassays against *Anopheles stephensi* studied by Kalayanasundaram and Das [65] revealed significant synergism between fenthion, a broad-spectrum organophosphate, and extracts of *Leucus aspara*, *Turnera ulmifolia*, *Vinca rosea*, *Clerodendron inerme*, *Pedalium murax* and *Parthenium hysterophorus*, with synergistic factors (SFs) of 1.31, 1.38, 1.40, 1.48, 1.61 and 2.23, respectively. In the larvicidal screening of 15 mangrove plant species, petroleum ether extract of *Rhizophora apiculata* stilt root was found to be most effective, with an LC_50_ value of 25.7 mg/l against *Culex quinquefasciatus* [66]. The synergism between this extract and pyrethrum, a botanical insecticide, was also recorded as reducing the LC_50_ of pyrethrum against *C. quinquefasciatus* larvae from 0.132 to 0.107 mg/l; also, the SF calculation of 1.23 followed the formula shown in this study [34, 35, 44]. The combined larvicidal action of *Solanum xanthocarpum* root extract and certain synthetic insecticides, namely fenthion, cypermethrin (a synthetic pyrethroid) and temephos (an organophosphate larvicide), were evaluated against *An. stephensi* [54] and *C. quinquefasciatus* [34]. The combined application of cypermethrin and *S. xanthocarpum* petroleum ether extract showed its synergistic action toward cypermethrin at all ratios. The most effective ratio was a binary 1:1 combination, showing an LC_50_ value and SF of 0.0054 ppm and 6.83, respectively, against *An. stephensi* [54]. While a 1:1 binary mixture of *S. xanthocarpum* and temephos acted antagonistically (SF = 0.6406), the *S. xanthocarpum*-fenthion combination (1:1) was observed to exhibit synergistic activity against *C. quinquefasciatus*, with an SF of 1.3125 [34]. Tong & Bloomquist [35] studied the effect of plant EOs on the toxicity of carbaryl, a wide-spectrum carbamate, and permethrin against *Ae. aegypti*. It was revealed that EOs of *Amyris balsamifera*, *Piper nigrum*, *Juniperus virginiana*, *Helichrysum italicum*, *Santalum album* and *Sesamum indicum* increased the toxicity of carbaryl against *Ae. aegypti* larvae, with SR values ranging from 1.0 to 7.0. Conversely, none of the EOs were toxic to adult *Ae. aegypti* and no synergistic effects were recorded in the EO-carbaryl combinations against this stage. PBO was used as the positive control that potentiated the toxicity of carbaryl against *Ae. aegypti* larvae and adults, with SR values of 4.9–9.5 and 2.3, respectively. Binary mixtures of permethrin and EO or PBO were investigated in only larvicidal activity. While the EO-permethrin mixtures acted antagonistically, the PBO-permethrin mixtures recorded synergistic interaction against *Ae. aegypti* larvae. However, the dose-response experiments and the SR estimation of the PBO-permethrin mixtures had not been performed. Although little work has been achieved on the synergistic, adulticidal activity of botanical-synthetic combinations against mosquito vectors, these findings lend support to the present results, which have opened up the prospects for synergist addition that not only decreases dose application but also increases insecticidal potency. Furthermore, the findings of this study demonstrate, for the first time, the significantly superior efficacy of synergizing permethrin toxicity with *C. rotundus* and *A. galanga* oils over PBO against both pyrethroid susceptible and resistant strains of *Ae. aegypti*. However, an unexpected finding from the synergism assay revealed that *C. verum* oil, which afforded the greatest adulticidal activity against both strains of *Ae. aegypti*, surprisingly provided an unsatisfactory effect with permethrin toxicity. Variability in the toxic actions and synergistic phenomena may be partly because of the influence of the variety of types and levels of bioactive principles in these oils.

Despite vigorous efforts to understand how efficacy enhancement can be brought about, the synergy mechanism is still not clearly understood. Possible reasons for distinct efficacy and synergistic potential could be the differences in the chemical compositions of the test product and mosquito susceptibility associated with the status and development of resistance. There was a difference in the constituents of the EOs tested in this study between the major and the minor compositions, in which some compounds have proved to exhibit repellent and toxic effects on various pests and disease vectors [61, 62, 64, 67, 68]. However, none of the main compounds characterized from *C. rotundus*, *A. galanga* and *C. verum* oils, such as cyperene, *β*-bisabolene and cinnamaldehyde, respectively, have been tested herein for adulticidal and synergistic activities against *Ae. aegypti*. Therefore, future investigations would be needed to isolate the active constituents present in each of the essential oils and elucidate their insecticidal efficiency and synergistic interactions against this mosquito vector. In general, insecticide activity depends on actions and counteractions between a toxicant and insect tissues, which are simplified into three stages: penetration of the insect integument as well as the membranes of the target organs, activation (= target site interaction) and detoxification of the toxicant [57, 69]. Therefore, the insecticide synergy that leads to enhancement of the potency of a toxicant combination needs at least one of these categories, for example, increased penetration, greater activation of the accumulated compound, or less detoxification of the active insecticidal constituent. Vigor tolerance, for example, delayed the cuticular penetration with a thickened cuticle layer and biochemical resistance, such as the enhanced metabolism of insecticides which was observed in some resistant strains of insects [70, 71]. The significant efficacy in potentiating permethrin toxicity of EO, particularly on PMD-R, is probably indicative of the addressing of insecticide resistance problems by interacting with resistance mechanisms [57, 69–71]. Tong & Bloomquist [35] supported the results of this study by showing synergistic interactions between EOs and synthetic pesticides against *Ae. aegypti*, with evidence of inhibitory activity in detoxifying enzymes, including cytochrome P450 monooxygenases and carboxylesterases, which are related prominently to the development of resistance to conventional pesticides. It is claimed that PBO also enhances good insecticide penetration, except as a metabolic inhibitor of cytochrome P450 monooxygenases, as supported by its use as the positive control in synergist studies [35, 72]. Interestingly, 1,8-cineole, which is one of essential constituents identified in *A. galanga* oil, was noted for its toxic effect on insect species [22, 63, 73] and has been reported for its combinations with synergistic bioactivity in several areas of research [74–77]. Furthermore, the penetration-enhancing effect of 1,8-cineole was recorded when combined with various pharmaceutical drugs, including curcumin [78], 5-fluorouracil [79], mefenamic acid [80] and zidovudine [81], either *in vivo* or *in vitro*. The probable role of 1,8-cineole in synergistic insecticidal activity is, therefore, not only as an active principle but also as a penetration enhancer. With greater synergism with permethrin, particularly on PMD-R, the synergistic effects of *A. galanga* and *C. rotundus* oils observed in this study are possibly a consequence of interactions with the resistance mechanism, that is, enhancing of permethrin penetration, increasing of activation of the accumulated compound, and inhibition of detoxifying enzymes such as cytochrome P450 monooxygenases and carboxylesterases. However, these aspects need further investigation in order to elucidate the specific role of EOs and their isolated compounds, both individually and in combinations, in the synergistic mechanism.

An increasingly high level of permethrin resistance in the main vector populations of Thailand was detected in 1977, and the use of permethrin was then largely substituted in later decades by use of other pyrethroid chemicals, particularly deltamethrin [82]. However, vector resistance to deltamethrin and other classes of insecticides is now extremely widespread throughout the country due to excessive and continuous use [14, 17, 83–86]. In order to resolve this issue, rotation or reuse of discarded insecticides that were effective previously and are less toxic to mammals, such as permethrin, has been recommended. Currently, permethrin resistance can still be detected in mosquito populations although its use has declined gradually in recent national public mosquito control programs. This may be due to mosquitoes being exposed to commercial household pest control products, mostly consisting of permethrin and other pyrethroids [14, 17]. Therefore, successful achievement regarding reuse of permethrin requires strategies to reduce vector resistance and increase implementation. Although none of the EOs tested individually in this study were as effective as permethrin, it was observed that their combined effect with permethrin can produce impressive synergistic results. This is a promising indication that interactions of EOs with resistance mechanisms, resulting in combinations of permethrin with EO, are more effective than the application of insecticides or EOs alone, specifically in PMD-R *Ae. aegypti*. The benefits of synergistic mixtures in increasing potency, despite the use of lower dosages applied in vector control, could possibly lead to improved resistance management and reduced costs [33, 87]. It is encouraging to note from these findings that EOs of *A. galanga* and *C. rotundus* have significantly greater efficacy in synergizing permethrin toxicity than PBO in both MCM-S and PMD-R strains, and are potential substitutes for conventional synergists.

The significant synergism of the selected EOs in enhancing adulticidal toxicity against PMD-R *Ae. aegypti*, particularly *A. galanga* oil with the extraordinary SR value of 1233.33, presents EOs as having a promising role as synergists for improving permethrin efficacy. This could encourage the use of a new active natural product whose joint action may prolong the use of highly effective mosquito control agents. It also sheds light on the possibility of EOs as alternative synergists for efficacious improvement of earlier or conventional insecticides in order to combat the existing problem of resistance in mosquito populations. Employment of readily available plants in mosquito control programs not only decreases dependence on imported and expensive materials, but also stimulates local efforts to enhance the public health system.

## Conclusions

These research findings have clearly thrown light on the significant synergizing actions that result from combining EOs and permethrin. The results emphasize the potential of EOs for use in mosquito control as a botanical synergist for improving permethrin efficacy against mosquitoes, particularly in resistant populations. Future studies on synergism bioassays of *A. galanga* and *C. rotundus* oils and their isolated compounds, combined with insecticides of either natural or synthetic origin against a variety of mosquito species and stages, and toxicity testing in non-target organisms are required for development and practical exploitation of EOs as viable alternative synergists.

## Abbreviations

DDT: Dichlorodiphenyltrichloroethane
CI: Confidence intervals
CMU: Chiang Mai University
EI: Electron ionization
GC-MS: Gas chromatography-mass spectrometry
LD: Lethal dose
MCM-S: Muang Chiang Mai-susceptible strain of *Aedes aegypti*
PBO: Piperonyl butoxide
PMD-R: Pang Mai Dang-resistant strain of *Aedes aegypti*
RI: Retention indices
RR: Resistance ratio
SR: Synergism ratio
ULV: Ultra-low volume
WHO: World Health Organization
